# UNRAQ—A Questionnaire for the Use of a Social Robot in Care for Older Persons. A Multi-Stakeholder Study and Psychometric Properties

**DOI:** 10.3390/ijerph18116157

**Published:** 2021-06-07

**Authors:** Slawomir Tobis, Agnieszka Neumann-Podczaska, Sylwia Kropinska, Aleksandra Suwalska

**Affiliations:** 1 Department of Occupational Therapy, Poznan University of Medical Sciences, 60-781 Poznan, Poland; 2 Chair and Department of Palliative Medicine, Poznan University of Medical Sciences, 61-245 Poznan, Poland; ar-n@wp.pl (A.N.-P.); skropins@ump.edu.pl (S.K.); 3 Department of Mental Health, Chair of Psychiatry, Poznan University of Medical Sciences, 60-572 Poznan, Poland; asuwalska@ump.edu.pl

**Keywords:** social robot, assessment tool, older adults, stakeholders, care

## Abstract

(1) Background: while there exist validated measures to assess the needs of older people, there are comparatively few validated tools to assess needs and requirements for the use of robots. Henceforth, the aim of the study is to present and validate such a tool. (2) Methods: The study group included 720 subjects (mean age 52.0 ± 37.0, 541 females) who agreed to fill the Users’ Needs, Requirements, and Abilities Questionnaire (UNRAQ). The validation part of the study included 125 persons. (3) Results: the acceptance of the robot was good in the whole group. The social functions were rated worse than assistive ones. A correlation was found between the scores of social and assistive functions. The respondents claimed that older adults were not prepared to interact with the robot and not very good at handling it, and were sceptical about their willingness to learn to operate the robot. The Cronbach alpha value for the whole questionnaire was 0.95 suggesting excellent internal consistency, and the ICC value of 0.88 represents excellent agreement; (4) Conclusions: We observed a good overall acceptance of the robot across the studied group. There is considerable demand for the use of a social robot in care for older people.

## 1. Introduction

With the rise of the digital age, it was only a question of time that modern technologies would be expected to accomplish complex tasks and reach the level of maturity required to be deployed in therapy and care. Among them, robotic technologies seem to be particularly predestined to be considered helpful in care for older people due to their potentially useful functions as well as the ability to take a humanoid shape that resembles a (human) caregiver and is more likely to be accepted by its older user [1].

It is also crucial to take into account that the process of ageing often leads to dependence on caregivers due to increasing functional limitations [2]. The rapid increase in the number of older people on a global scale thus has an impact on the needs of services. The difference between the (increasing) number of older subjects in need of support and the number of caregivers available in the system is commonly referred to as a “care gap”. Robots have been proposed as one of the forms of assistive devices that can help bridge the widening gap between the need and supply of healthcare services. These devices promise adequate support for older persons’ independence by preventing functional decline and improving well-being [3]. The idea of using a robot in care has been studied for decades [4], with conclusions related to the design of robots, their proposed roles, ethical issues, and human-robot interaction in the first place [5,6,7,8].

Older people have been shown to be prepared to accept assistive devices in healthcare and housing when the aim is to help them maintain their independence [9,10], particularly so when there exists a perceived need for the device [11].

The failure of at least some of the telemedicine projects can be partly attributed to not correctly assessing needs and requirements [12]. Cautiously assessing needs and mapping these to the technology to be provided can result in higher acceptance rates [13]. This can be achieved by conducting a survey prior to the implementation [12]. It is also imperative to take into consideration the opinions of various stakeholders, also including the formal/informal caregivers of the older subjects.

The approach to implementation and provision of maintenance for the technology to be deployed are critical factors for the user’s acceptance and satisfaction, considering that telehealth and telecare technologies are not and never will be “plug and play” or “one size fits all” [14]. The solutions to be implemented must thus be tailored and customised to the user’s needs and preferences [10].

While there exist validated measures to assess the needs of older people [15], there are comparatively few validated tools to assess needs and requirements for the daily-life use of robots, which is one of the reasons that the quality of studies performed in this area is mostly low to moderate [16,17].

There are also some validated measures of responses and acceptance of robots (such as Negative Attitudes towards Robots Scale [18], Robotic Social Attributes Scale [19], Ethical Acceptability Scale [20], Technology-Specific Expectation Scale [21], Frankenstein Syndrome Questionnaire [22], Multi-Dimensional Robot Attitude Scale [23] or Attitudes Towards Social Robots Scale [24]), yet they do not focus on the care needs of older adults [25]. Valid and reliable tools to assess older persons’ needs and requirements regarding the properties and functions of a robot to be deployed in care are thus needed.

Henceforth, the aim of the study was to present and validate the Users’ Needs, Requirements and Abilities Questionnaire (UNRAQ)—a tool that can be used to collect the data about the use of a social robot in care for older persons from various perspectives.

## 2. Materials and Methods

The research project using the UNRAQ questionnaire was approved by the Bioethics Committee of Poznan University of Medical Sciences, Poland (No. 711/18).

### 2.1. Participants

The study group included conveniently available individuals who agreed to share their opinions on the use of robots to support older people living at home. First, participants were recruited during a series of lectures and workshops run by members of the team. Further on, snowball sampling was used.

The study group included 720 subjects. The persons who participated in the validation part (125 people) expressed their opinions on the use of a robot in care for older people twice, two weeks apart.

### 2.2. Procedure

The Users’ Needs, Requirements and Abilities Questionnaire (UNRAQ) was developed to collect data related to the robot requirements as a part of the ENRICHME project [26]. Initial results obtained with this tool have already been presented [27,28]; however, the UNRAQ has so far not been validated for its reliability. The original questionnaire was modified for better comprehensiveness; the modification removed two statements: one which was difficult to understand, and one which partially repeated content from two other statements. The remaining statements were structured into coherent blocks.

The first part of the UNRAQ characterises the participant (by data such as age, sex, level of education, being a caregiver of an older person, familiarity with technology and ability to operate a computer). The following quantitative part is divided into four areas, which all consist of a series of statements. The participants are asked to rate each of these statements by expressing their level of agreement (or disagreement) based on a 5-point Likert scale (1—I strongly disagree, 2—I partially disagree, 3—I neither agree nor disagree, 4—I partially agree, 5—I strongly agree). Scores 4–5 are considered positive. Thanks to such a structure, the results can be presented as means and standard deviations (SD). The participant can also comment in a free form on any statement in an extra box provided next to it.

The particular UNRAQ areas are:

- interaction with the robot and technical issues (10 statements),
- assistive role of the robot (13 statements),
- social aspects of using the robot (6 statements),
- ethical issues (5 statements).

The UNRAQ ends with a Creativity Box that presents the participant with a possibility to express any comments, ideas, suggestions, or observations that come to their mind and which are not covered by the statements of the questionnaire.

Before the survey, the participants were presented with a picture of a humanoid robot to obtain a realistic image of the robot concept; additionally, a research team member was available to clarify emerging doubts on an ongoing basis.

### 2.3. Statistical Analysis

Statistical analysis was performed with the STATISTICA (StatSoft, Kraków, Poland) and SPSS (IBM, Warszawa, Poland) software. Variables were expressed as percentages, frequencies, means ± SD, and medians. The normality of data distribution was examined with the Shapiro-Wilk’s test. Due to lack of normality, comparisons between two paired groups of data were performed with the Wilcoxon test and between two unpaired groups—with the Mann-Whitney test. Differences in the distribution of quality variables between two groups were assessed with the χ^2^ test with Yates correction due to the small sample size. *p* < 0.05 was considered statistically significant.

Internal consistency and test-retest statistical methods for reliability were used to validate the UNRAQ and prove its psychometric properties. For internal consistency, the Cronbach’s alpha value was calculated based on the results of the baseline UNRAQ. For the evaluation of results, the George and Mallery rating score was used (≥0.9: excellent, <0.9–≥0.8: good, <0.8–≥0.7: acceptable, <0.7–≥0.6: questionable, <0.6–≥0.5: poor, and ≤0.5: unacceptable [29]).

Test-retest reliability was assessed with the Intraclass Correlation Coefficient (ICC) based on the two sets of UNRAQ results obtained in the studied subjects (single measure, absolute agreement) [30]. According to the recommendation by Cicchetti and Sparrow [31], ICC ≥ 0.75 indicates excellent agreement, 0.60 to 0.74—good, 0.40 to 0.59—fair to moderate, and <0.40—poor agreement.

## 3. Results

Seven hundred and twenty persons participated in the study (mean age 52.0 ± 37.0 (50; 19–91), including 273 people (37.9%) who were at least 60 years old. Among the respondents, there were 541 women (75.1%). Three hundred twenty-two subjects had a degree of higher education (45.7%), 474 (66.5%) people declared that they were familiar with technology, and 328 (45.7%) were caregivers of older persons. In the older subgroup, 63 persons had a degree of higher education (23.1%), 136 (49.8%) subjects declared being familiar with technology, and 82 (30.0%) being a caregiver to an older person. The mean age of this subgroup was 72.6 ± 8.3 years.

Based on the UNRAQ results, the acceptance of the robot by the whole group was good. The results for every individual statement are presented in Table 1.

The highest scores were found in domain B (assistive role of the robot; mean for the domain: 4.6 ± 0.7; median: 4.8; 1–5); on average, 90.3% of participants agreed with statements in this area. The functions related to safety and health aspects (B1—*The robot should increase the safety of the elderly home: for example, locking doors, detecting leaking gas,* etc., B2—*The robot should help the elderly to preserve their memory function, e.g., by playing memory games with them*, B9—*The robot should remind the elderly about medication*, and B12—*The robot should call the centre in case of emergency*) were rated the highest. On the other hand, functions related to eating were scored the lowest (B4—*The robot should provide advice about a healthy diet* and B7—*The robot should monitor the amount of food and fluid intake of the owner*).

**Figure 1:**
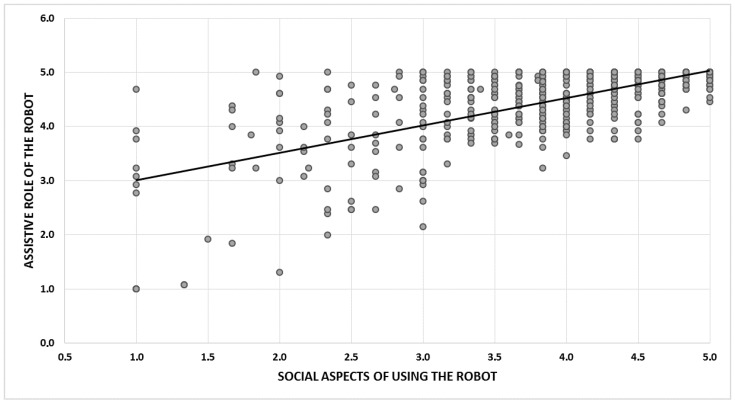
Relationship between the assessment of social and assistive functions in 720 people.

The social aspects of using the robot (domain C) were rated significantly worse than the assistive ones (domain B): 4.1 ± 0.9 (4.2) vs. 4.6 ± 0.7 (4.8); *p* < 0.001); on average, only 76.5% of people agreed with statements from this area. This result is significantly lower than that for domain B (*p* = 0.000). However, a correlation was found between the summarised scores of the social and assistive functions (Figure 1; r = 0.662; *p* < 0.0001).

Within domain A, the respondents claimed that older adults are not prepared to interact with the robot and are not very good at handling it (2.4 ± 1.2 [2.0] and 2.6 ± 1.2 [2.0], respectively). In addition, they also negatively assessed the prospect that that older people will want to learn to operate the robot, although they scored this statement higher than the previous two (3.2 ± 1.2 [3.0], *p* < 0.001).

The largest percentage of respondents believed that the robot should primarily be a useful device for an older person (something to be used when needed with no other interaction); 623 positive answers—86.8% of all respondents. Comparably often, the robot was expected to be an assistant of the older person; 604 positive answers—84.1% of all respondents. The usefulness of the robot as a companion of the older person was assessed much worse than the above two functions; 478 positive responses—66.8% (*p* < 0.001).

In domain D, higher acceptance of statement D3 (it is acceptable that the robot informs a family member or caregiver about the older person’s behaviour/health problems) was observed than that of the remaining ones (*p* < 0.01).

As part of their free statements, older persons pointed out that “such a robot will certainly be easy to use so that it is [accessible] for everyone” (woman, 72). It was also crucial for them that someone at the beginning explained the operation and, possibly, clarified the emerging doubts “it’s good that someone young comes, connects everything, demonstrates, and then comes as long as it takes until everything is easy and obvious for the service” (man, 78).

### 3.1. Parameters Relevant to the Answers Provided in the UNRAQ Questionnaire

The respondents’ gender was not significant for the answers given.

Regarding the robot as *a useful device of the elderly person*, the frequency of giving positive answers was higher in respondents with higher education (89.8% vs. 84.5%; OR 1.612 [CI 1.002–2.594]; *p* < 0.05) and those declaring familiarity with technology (89.2% vs. 82.0%; OR 1.701) [CI 1.074–2.697]; *p* < 0.05), but also lower in subjects being caregivers (83.2% vs. 90.0%; OR 0.503 [CI 0.317–0.796]; *p* < 0.01).

As far as other roles of the robot are concerned, the only parameter significant for the higher frequency of positive scores of the assistant function was being familiar with technology (87.3% vs. 77.8%; OR 1.057 [CI 1.301–2.942]; *p* < 0.01). For the usefulness of the robot as a companion of the older person, subjects who were caregivers gave positive answers less frequently (62.2% vs. 70.7%; OR 0.681 [CI 0.498–0.931]; *p* < 0.05).

For the remaining part of domain A, the most important parameter influencing the differentiation of the answers expressed was respondents’ age. Older people (60 and over) more often gave positive answers to the following statements:

- A5 (*The elderly are able to manage with the robot*): 34.9% vs. 21.9%; OR 1.920 (CI: 1.372–2.686),
- A6 (The elderly want to increase their knowledge about the robots to be able to operate them): 56.8% vs. 37.5%; OR 2.187 (CI: 1.609–2.972),
- A9 (The elderly should be able to choose the functions of the robot they want to use and disable other ones): 83.2% vs. 72.0%; OR 1.922 (CI: 1.316–2.805),
- A10 (If the robot has been switched off by the owner, it should reactivate automatically (after a specific period) so that it is not forgotten in off mode: 89.4% vs. 80.3%; OR 2.062 (CI: 1.315–3.235).

In domains B and C, the most important parameter relevant to the answers provided was *being familiar with the technology*. People declaring familiarity more often gave positive answers to the following statements:

- B2 (The robot should help the elderly to preserve their memory function, e.g., by playing memory games with them): 94.3% vs. 87.9%; OR 2.276 (CI: 1.314–3.942),
- B3 (The robot should encourage and guide the elderly to perform physical exercises): 93.5% vs. 84.5%; OR 2.612 (CI: 1.575–4.330),
- B4 (The robot should provide advice about a healthy diet): 86.9% vs. 79.1%; OR 1.758 (CI: 1.166–2.650),
- B9 (The robot should remind the elderly about medication): 97.1% vs. 92.0%; OR 2.851 (CI: 1.403–5.792),
- B13 (The robot should help the owner to find lost objects (e.g., glasses. keys): 93.9% vs. 89.5%; OR 1.793 (CI: 1.025–3.137),
- C2 (The robot could encourage the elderly to enhance their contacts with friends): 82.4% vs. 74.9%; OR 1.571 (CI: 1.078–2.289),
- C3 (The robot should initiate contacts with others (calling friends, initiating Skype conversations): 78.4% vs. 69.8%; OR 1.578 (CI: 1.108–2.245),
- C4 (The robot should have entertainment functions (e.g., gaming partner, reading aloud, or playing music): 88.8% vs. 82.9%; OR 1.645 (CI: 1.058–2.557).

In the domain D, for the statements

- D2 (*The elderly person should be able to send the robot to its place/docking station and keep it staying there*)—persons being caregivers gave less often positive scores: 68.8% vs. 78.5%; OR 0.606 (CI: 0.433–0.848),
- D3 (It is acceptable that the robot informs a family member or caregiver about the older person’s behaviour/health problems)—persons familiar with technology gave more often positive scores: 89.0% vs. 81.2%; OR 1.882 (CI: 1.220–2.905),
- D5 (*It is acceptable that the robot will have much information about the user (social, medical, others*)—persons with higher education gave more often positive scores: 80.1% vs. 72.3%; OR 1.542 (CI: 1.083–2.197).

### 3.2. Psychometric Properties

The reliability study included 125 subjects. The Cronbach alpha value for the whole questionnaire was 0.95, suggesting excellent internal consistency. In the analysis of values for each domain, they were all above the cut-off point for enough quality (0.70), with the lowest values for *Ethical issues* and *Interaction with the robot and technical issues* (0.77 for both). The ICC values of 0.88 for the whole questionnaire, and ranging from 0.81 to 0.93 for the domains, were found, which represents excellent agreement—both for individual domains and the questionnaire as a whole (Table 2).

## 4. Discussion

The growing care gap requires taking adequate actions to secure the sustainability as well as quality of care and support for older persons, now and even more so in the future. One of the possible approaches in this regard is the use of socially assistive robots in those areas in which it is viable and desired by potential older users. Robots are more complex than other technological devices, and such are also the factors of their acceptance [32]. For successful introduction of the robot, it is essential to study the needs and requirements as well as the factors which affect the acceptance and readiness to use the robot in somebody’s daily life, possibly considering the perspective from a range of stakeholders, such as older persons, their families, and caregivers [3]. We present the results of a study for which a dedicated measurement tool was created (the UNRAQ). Our questionnaire proved to have good psychometric properties.

The robot was perceived as a useful device in the first place, that is, something that can be used when needed, which is in line with the observation by de Graaf et al. that the purpose of the robot must be clear for its successful acceptance [33]. This function scored the highest among participants familiar with technology. However, the importance of the category of “usefulness” has also been found in long-term studies which focused on the acceptance factors of service robots for domestic use [34,35].

The worst-rated function of the robot was being a companion of the older person, which seems to indicate that it is still difficult to imagine a machine taking over the role of a human. Our observation is similar to those from other studies suggesting that people do not value the social behaviour of the robots, at least at this stage of social robot presence in society [36,37]. Those who declared being caregivers rated the robot worse, both as a companion and a useful device, which may result from the belief that human care cannot be fully substituted with a robotic one [38]. This view has been confirmed in the ethics part of the UNRAQ: for the statement that the user should be able to send the robot to its place/docking station and keep it staying there, we observed lower scores from the caregivers versus those of older adults, which may also reflect a difference in the attitude to the question of controlling the robot.

The assistive functions were scored high, particularly those related to health and safety (a phenomenon also found in the literature [39]), which may be an indication of unmet needs or fears present in these areas. The social functions obtained less positive scores—again, possibly due to the conviction that a human cannot be replaced by a robot in social relationships. Yet, those who were more optimistic about the use of robots in care for older people generally showed a broader acceptance. This was particularly the case in the subgroup declaring familiarity with technology, which may have unleashed additional creativity, enabling them to better imagine the possible applications of the robot. This subgroup seems to trust the technology more, as the scores of the statement “*It is acceptable that the robot informs a family member or caregiver about the older person’s behaviour/health problems*” show. Similar findings were presented by Khaksar et al. for the broad category of assistive technologies [40].

The preparedness of older persons to operate the robot and the ability to cope with it were viewed as insufficient, whereas the opinions of the older adults themselves were better in this regard, similarly to published studies [27,41]. Older adults also significantly more often expressed the willingness to *increase their knowledge about the robot to be able to operate them*. This may be viewed as a sign of openness towards the robot, provided the users receive competent training and support in this regard, which is consistent with the studies of Bedaf et al. [10,42]. Notable differences between studied subgroups in judging the preparedness and ability to cope with the robot indicate reservations of the younger versus the older ones (also appearing in the free text comments). Additionally, the statement about the possibility of customisation of the robot’s functions by its user was scored high (higher by older subjects than caregivers), which points out that older persons have a comparatively high level of reflection on the potential role of the robot in their life and seem more independent in their decisions than believed by the caregivers.

On the other hand, a high score of the statement “If the robot has been switched off by the owner, it should reactivate automatically (after a specific period), so that it is not forgotten in off mode” indicates that there are fears on both sides (more on the side of the older participants) that the ability to operate the robot may be impacted by the diminished cognitive ability of its user (similarly to the observations of Wang et al. [43]).

The results of a study like ours may be influenced by cultural context; further re-search in this area could contribute to establishing the degree of its impact [44]. More-over, robotic technologies are constantly progressing; among those related to the subject of the study, two stand out: the development of affective sensing and adaptation (enabling the machine to provide a kind of emotional response to its user) [45], and multi-face and gesture recognition (thanks to advances in sensors and cloud processing) [46], potentially enhancing remote human services. Getting increasingly familiar with such technologies may also affect the perception of the robot.

Our study has some limitations—its cross-sectional nature (no longitudinal data) and lack of direct human-robot interaction with study participants. Nonetheless, its strength is the large study group composed of various stakeholders, thanks to which, a diversified perspective on the matter can be obtained.

## 5. Conclusions

We observed a good overall acceptance of the robot across the studied group. Our study demonstrates that there is considerable demand for the use of a social robot in care for older people. The UNRAQ showed good psychometric properties.

## Figures and Tables

**Table 1:** Results for the individual UNRAQ statements.

Domain	Statement	Mean ± SD Median	Positive Answers
A: INTERACTION OF THE ROBOT AND TECHNICAL ISSUES	A1 The robot should be a companion of the elderly person	3.7 ± 1.3 (4.0)	478; 66.8%
	A2 The robot should be an assistant of the elderly person	4.2 ± 1.1 (5.0)	604; 84.1%
	A3 The robot should be a useful device of the elderly person (something to be used when needed, with no other interaction)	4.4 ± 1.0 (5.0)	623; 86.8%
	A4 The elderly are prepared to interact with a robot	2.4 ± 1.2 (2.0)	150; 21.0%
	A5 The elderly are able to manage with the robot	2.6 ± 1.2 (2.0)	192; 26.8%
	A6 The elderly want to increase their knowledge about the robots to be able to operate them	3.2 ± 1.2 (3.0)	322; 44.8%
	A7 The robot should instruct the elderly person what to do in case of problems with its operation	4.5 ± 1.0 (5.0)	616; 86.0%
	A8 The robot should be customisable (adjusted to individual user preferences and needs)	4.6 ± 0.8 (5.0)	647; 90.0%
	A9 The elderly should be able to choose the functions of the robot they want to use and disable other ones	4.1 ± 1.1 (4.0)	548; 76.2%
	A10 If the robot has been switched off by the owner it should reactivate automatically (after a specific period). so that it is not forgotten in off mode	4.4 ± 1.0 (5.0)	603; 83.8%
B: ASSISTIVE ROLE	B1 The robot should increase the safety of the elderly home: e.g., locking doors. detecting leaking gas etc.	4.6 ± 0.8 (5.0)	653; 90.8%
	B2 The robot should help the elderly to preserve their memory function e.g., by playing memory games with them	4.6 ± 0.8 (5.0)	662; 92.2%
	B3 The robot should encourage and guide the elderly to perform physical exercises	4.5 ± 0.9 (5.0)	651; 90.5%
	B4 The robot should provide advice about a healthy diet	4.3 ± 1.0 (5.0)	608; 84.4%
	B5 The robot should monitor the environment (temperature, humidity) and suggest air conditioning adjustment or windows opening	4.5 ± 0.9 (5.0)	640; 89.0%
	B6 The robot should measure physiological parameters (blood pressure, heart rate, body temperature) of the elderly person	4.6 ± 0.8 (5.0)	653; 90.9%
	B7 The robot should monitor the amount of food and fluid intake of the owner	4.3 ± 1.0 (5.0)	593; 82.5%
	B8 The robot should remind the elderly about appointments	4.5 ± 0.9 (5.0)	642; 89.4%
	B9 The robot should remind the elderly about medication	4.7 ± 0.7 (5.0)	686; 95.4%
	B10 The robot should remind about meals times. drinks	4.5 ± 0.9 (5.0)	635; 88.3%
	B11 The robot should observe the behaviour of the elderly person to detect falls or changes due to illness	4.6 ± 0.8 (5.0)	660; 91.9%
	B12 The robot should call the centre in case of emergency	4.8 ± 0.7 (5.0)	688; 95.8%
	B13 The robot should help the owner to find lost objects (e.g., glasses, keys)	4.6 ± 0.8 (5.0)	663; 92.5%
C: SOCIAL ASPECTS	C1 The robot could decrease the sense of loneliness and improve the mood of the elderly person	3.9 ± 1.2 (4.0)	523; 72.9%
	C2 The robot could encourage the elderly to enhance their contacts with friends	4.1 ± 1.1 (4.0)	575; 80.1%
	C3 The robot should initiate contacts with others (calling friends, initiating Skype conversations)	4.0 ± 1.2 (4.0)	544; 75.8%
	C4 The robot should have entertainment functions (e.g., gaming partner, reading aloud or playing music function)	4.4 ± 1.0 (5.0)	626; 86.9%
	C5 The robot should detect the owner’s mood (facial expression)	4.0 ± 1.1 (4.0)	531; 74.0%
	C6 The robot should accompany the owner in everyday activities (watching TV, preparing meals)	3.8 ± 1.2 (4.0)	497; 69.1%
D: ETHICAL ISSUES	D1 The elderly person should have control over the robot	4.1 ± 1.1 (4.0)	546; 76.4%
	D2 The elderly person should be able to send the robot to its place/docking station and keep it staying there	4.1 ± 1.1 (4.0)	532; 74.0%
	D3 It is acceptable that the robot informs a family member or caregiver about the older person’s behaviour/health problems	4.4 ± 0.9 (5.0)	623; 86.5%
	D4 The elderly person should be able to switch off the robot in specific situations (friends’ visits, privacy reasons etc.)	4.2 ± 1.1 (5.0)	552; 76.7%
	D5 It is acceptable that the robot will have much information about the user (social, medical, others)	4.0 ± 1.1 (4.0)	547; 76.1%

**Table 2:** The Cronbach Alpha and the Intraclass Correlation Coefficients (ICC) for the domains of the UNRAQ (*n* = 125).

Domains		Cronbach Alpha	ICC
A	Interaction with the robot and technical issues	0.77	0.81
B	Assistive role of the robot	0.94	0.93
C	Social aspects of using the robot	0.87	0.90
D	Ethical issues	0.77	0.82

## Data Availability

The data presented in this study are available from the corresponding author upon reasonable request.
